# Repetitive Concussions in Adolescent Athletes – Translating Clinical and Experimental Research into Perspectives on Rehabilitation Strategies

**DOI:** 10.3389/fneur.2015.00069

**Published:** 2015-04-02

**Authors:** Bridgette D. Semple, Sangmi Lee, Raha Sadjadi, Nora Fritz, Jaclyn Carlson, Carrie Griep, Vanessa Ho, Patrice Jang, Annick Lamb, Beth Popolizio, Sonia Saini, Jeffrey J. Bazarian, Mayumi L. Prins, Donna M. Ferriero, D. Michele Basso, Linda J. Noble-Haeusslein

**Affiliations:** ^1^Department of Neurological Surgery, University of California San Francisco, San Francisco, CA, USA; ^2^Department of Medicine, Royal Melbourne Hospital, University of Melbourne, Parkville, VIC, Australia; ^3^Kennedy Krieger Institute, John Hopkins University, Baltimore, MD, USA; ^4^San Francisco State University Graduate Program in Physical Therapy, University of California San Francisco, San Francisco, CA, USA; ^5^School of Medicine and Dentistry, University of Rochester Medical Center, Rochester, NY, USA; ^6^Department of Neurosurgery, University of California Los Angeles David Geffen School of Medicine, Los Angeles, CA, USA; ^7^Department of Pediatrics, University of California San Francisco, San Francisco, CA, USA; ^8^Department of Neurology, University of California San Francisco, San Francisco, CA, USA; ^9^School of Health and Rehabilitation Sciences, Ohio State University, Columbus, OH, USA; ^10^Department of Physical Therapy and Rehabilitation Sciences, University of California San Francisco, San Francisco, CA, USA

**Keywords:** concussion, adolescent, athletes, experimental models, behavior, pathology, rehabilitation, exercise

## Abstract

Sports-related concussions are particularly common during adolescence, a time when even mild brain injuries may disrupt ongoing brain maturation and result in long-term complications. A recent focus on the consequences of repetitive concussions among professional athletes has prompted the development of several new experimental models in rodents, as well as the revision of guidelines for best management of sports concussions. Here, we consider the utility of rodent models to understand the functional consequences and pathobiology of concussions in the developing brain, identifying the unique behavioral and pathological signatures of concussive brain injuries. The impact of repetitive concussions on behavioral consequences and injury progression is also addressed. In particular, we focus on the epidemiological, clinical, and experimental evidence underlying current recommendations for physical and cognitive rest after concussion, and highlight key areas in which further research is needed. Lastly, we consider how best to promote recovery after injury, recognizing that optimally timed, activity-based rehabilitative strategies may hold promise for the adolescent athlete who has sustained single or repetitive concussions. The purpose of this review is to inform the clinical research community as it strives to develop and optimize evidence-based guidelines for the concussed adolescent, in terms of both acute and long-term management.

## Introduction

Sports-related concussions are common during adolescence, a time when approximately half of all high-school students participate in sports in the United States (US) (1). Concussions account for 5–13% of all reported sports injuries in high-school-aged athletes (1–3). In high-school football, an estimated 5.6% of players sustain a concussion in a given season (4), although the actual incidence may be closer to 15% due to under-reporting (5).

There is increasing evidence that the adolescent, defined by the World Health Organization as between the ages of 10 and 19 years, may have poorer outcomes after concussions compared to older athletes (6–8). Importantly, repetitive concussions to the brain during maturation may result in both acute and long-term complications (9). Moreover, even subtle cognitive deficits may have profound consequences on both academic and athletic performance and social integration. There has been considerable attention to identifying risk factors for concussions for professional athletes in contact sports, and a burgeoning interest in injuries sustained at a younger age. Most recently, the Institute of Medicine (IOM) and National Research Council released a report titled “Sports-Related Concussions in Youth: Improving the Science, Changing the Culture,” which considers risk factors, screening, detection, treatment and management recommendations, and long-term consequences of repetitive concussions in the developing brain (10). In addition, an updated consensus report from the Fourth International Conference of Concussion in Sports (11) and evidence-based guidelines for the management of sports concussions have recently been published (12). These reports provide an important foundation for understanding the consequences of concussion and acceptable practices for medical management.

Here, we provide an overview of adolescent concussions from a clinical perspective, address the utility of rodent models in understanding the pathobiology and behavioral consequences of concussions, and discuss how repetitive concussions may alter injury progression and neurobehavioral outcomes. In addition, we consider how best to optimize recovery, recognizing that optimally timed, exercise-based rehabilitative strategies may hold promise for the adolescent who has sustained single or repetitive concussions.

## Definition and Diagnosis

In this review, we define concussion as a subset of traumatic brain injury (TBI), in line with the consensus report from the Fourth International Conference of Concussion in Sports (11). A concussion, or mild TBI, is a complex process triggered by a biomechanical insult to the brain, which is typically not associated with the presence of early structural damage on standard neuroimaging (13, 14). Suspected diagnosis is primarily based upon reported symptoms, which may be evaluated together with neuroimaging to rule out a more severe injury, as evidenced by skull fracture or intracranial hemorrhage (15). Symptoms may include headache, fatigue, nausea, dizziness, difficulty concentrating, sleep disturbance, sensitivity to light and/or noise, balance problems, irritability, anxiety, and depression (1, 16). Among high school and college athletes, the most common symptom is headache (1, 17). A brief loss of consciousness (LOC) has previously been considered a hallmark of concussion (18); however, this symptom is no longer considered a defining feature as it presents in <10% of all concussions (4, 11, 17).

Most of the neurocognitive assessments available to assist in identification of a suspected concussion have been developed for adults (12). For children and adolescents over 12 years of age, the Sports Concussion Assessment Tool version 3 (SCAT3) was recently adapted from SCAT2, and the new Child-SCAT3 has been tailored for concussed children aged 5–12 years (11). Based upon data from experimental models (see Section “Pathology after Experimental Concussions at Adulthood”), single and repetitive concussions may result in acute neuropathology including axonal injury, microbleeds, and glial activation, all of which may be sources of biomarkers. Biomarkers with high sensitivity may thus prove useful to both detect pathology, establish baseline physiology, and track recovery after concussions (10, 19). As ongoing studies attempt to evaluate the potential benefit of rehabilitative interventions based largely upon the subjective self-reporting of symptoms [e.g., see Ref. (20)], biomarkers may provide a more objective measure. Further, in the context of individualized medicine, biomarkers may ultimately predict those who are at greater risk than others for developing long-term problems when exposed to repetitive concussions. State-of-the-art neuroimaging techniques such as single photon positron emission tomography, diffusion tensor imaging, functional magnetic resonance imaging (fMRI), nuclear magnetic resonance spectroscopy (NMRS), and high-definition fiber tracking also show promise for future utility in the assessment and detection of sports-related concussions with increased sensitivity (21–25).

The severity of concussion symptoms can vary and may take hours to days to manifest, particularly in children. Fortunately, the effects of a single concussion are typically transient, with acute neurological dysfunction resolving within 7–10 days in most adult athletes, and within 1 month in most younger athletes (11, 26). However, a subset of patients shows sub-acute or chronic symptoms including sleep dysregulation, cognitive deficits, and emotional disturbances (27, 28). Such persistent post-concussive symptoms affect between 2 and 11% of concussed patients more than 1 month after injury (27). Among children and adolescents, a range of 11–29% of patients report persistent symptoms up to 1 month after mild traumatic brain injuries, including single and repetitive concussions (28–32). A recent prospective cohort study of 335 young concussed athletes (8–23 years old) exhibited symptoms for an average of 43 ± 53 days (33). Emerging evidence that both metabolic dysfunction (34–36) and disruption of functional connectivity (23, 37) persist for several weeks to months after repetitive concussive insults, suggests that recovery to normal brain health may be prolonged even after the resolution of clinical symptoms.

## Epidemiology and Etiology

Youths typically engage in organized sports with increased frequency relative to adults, and thus sustain the majority of the sports-related concussions (38, 39). In fact, of the ~23,000 football-related, non-fatal TBIs resulting in emergency department visits annually in the US, almost 90% occur in adolescents aged 5–18 years (40). Among 15–24 year olds, sports-related head impacts are second only to road trauma as the most common cause of TBI (41). In contrast, concussions sustained during childhood, prior to 10 years of age, more often result from non-sports-related falls in the home, school, or playground (42). Over the past decade, the incidence of concussions reported by high-school students has increased by ~15% annually (43). It is unclear, however, whether this figure reflects a true increase in events, or a rise in reporting prompted by greater awareness.

Within the adolescent population, concussions occur most commonly in full-contact sports (e.g., football, soccer, ice hockey, and lacrosse) compared to non-contact sports (e.g., volleyball and swimming) (1, 44). Participation in football is responsible for the majority of sports-related concussions in the US (45, 46), likely due in part to its large number of participants, particularly at the high-school level (1, 2, 47). The most common injury mechanisms are player–player contact (70%) and falls (1). Concussion rates are typically higher during competition than in practice (48, 49).

By the start of high school, 53% of student athletes have reported a history of concussion (6). However, the actual incidence of sports-related concussions during adolescence may, in fact, be higher than reported. In a confidential survey across 20 schools in Wisconsin, 15% of high-school football players sustained a concussion during the previous football season; however, only 44% of these athletes reported their injury (5). Under-reporting may result from a lack of recognition by the athlete, coaches, trainers, or other medical personnel; not thinking the injury was serious enough to warrant a report; or fear of removal from participation (5, 50). On the contrary, over-reporting may also occur when athletes are asked to reflect on past events.

Gender differences in both the incidence and outcomes of concussions have also been reported. Comparison between the sexes is complicated by different numbers of males and females who play specific sports, as well as potential differences in baseline verbal and visual neuropsychological test measures during adolescence (51). However, reviews have repeatedly suggested that high school and college-aged female athletes are at higher risk of concussion, sustaining more injuries than males (3, 52). Further, females report a greater number and severity of symptoms, and show poorer outcomes, requiring a longer duration to recover (14, 52–56). In high-school sports played by both sexes (e.g., soccer, baseball, and basketball), concussions represented a greater proportion of total injuries in girls (3). The mechanisms underlying this gender bias are unclear, and may in part relate to biological differences that are compounded by cultural distinctions in how concussions are reported and managed (57).

### Repeated concussions in sports

The risk of a subsequent concussion is elevated in athletes with a history of previous concussions (44, 58, 59). Among high-school football players in the US, between 12 and 32% of those sustaining a concussion report a prior sports-related concussion either in the same season or the one prior (1, 4, 60, 61). In collegiate football athletes, between 6 and 36% have reported multiple concussions (6, 62).The brain is hypothesized to be more susceptible to subsequent injury after a concussion, such that sustaining a second impact while recovering from the first may result in prolonged and/or more severe symptoms (10, 17, 63). High school and amateur athletes who sustained two or more concussions exhibit greater impairment on neuropsychological and memory tests than athletes with a history of only a single concussion (60, 64, 65). Similarly, a recent multi-center study of over 600 high-school athletes found significantly higher ratings of cognitive, physical, and sleep-related concussion symptoms in athletes with a history of two or more concussions, compared to athletes with one or no previous concussions (66).

There is emerging evidence that repetitive concussions in both adults and adolescents may yield persistent neurocognitive changes (14, 65). Repetitive concussions have been implicated as a contributing factor to neurodegenerative conditions including chronic traumatic encephalopathy (67, 68), post-traumatic stress disorder (69), substance abuse (70), anxiety, and depression (69, 71). However, there remains a paucity of prospective or longitudinal studies examining repetitive concussions and long-term neurodegeneration. As such, there is insufficient information to determine if there is a causal relationship (72, 73). Together with evidence that concussed high-school athletes typically present with more severe cognitive deficits compared to concussed adults (6, 74, 75), the potential for long-term consequences after cumulative injuries highlights the need for a greater understanding of how the adolescent brain responds to concussions.

### Age-related effects of concussions

It is now widely accepted that the human brain continues to mature throughout childhood and adolescence, with considerable structural and functional changes ongoing well into the third decade of life (76, 77). However, despite the prevalence of sports-related concussions among adolescent-aged athletes, only a few studies have explored whether the adolescent brain shows age-dependent vulnerability to concussive insults; or, conversely, enhanced capacity for plasticity and recovery.

When comparing high school and collegiate athletes who have experienced sports-related concussions, the former performed more poorly on serial neuropsychological tests at 3, 5, and 7 days after injury (6), and took longer to return to neurocognitive and symptom-free baselines compared to older athletes (8). Average times to normalization of neurocognitive abilities in high-school students have been reported as 10–14 days, compared to 5–7 and 3–5 days in collegiate and professional athletes, respectively (78, 79).Together, these studies suggest that adolescents may be particularly susceptible to the consequences of concussive injury, although there remains a paucity of studies evaluating the recovery patterns of participants under the age of 15 years (14).

## Experimental Models of Single and Repeat Concussive Brain Injury in Rodents

While there is growing awareness of the adverse consequences of repeated concussive injuries, the exact mechanisms underlying these injuries remain unclear, as are the factors that (i.e. ’as are the factors that contribute…’) contribute to persistent symptomatology in some patients. Animal models, mimicking aspects of repetitive concussions, have served as a platform for monitoring both acute and long-term behavioral outcomes after injury, and for understanding the associated cellular and molecular mechanisms. In this section, we briefly review the published literature regarding experimental models of repetitive concussions in adult rodents and the resulting behavioral outcomes, and consider the few studies that have examined injury at an adolescent age.

### Injury parameters

Animal models of concussion typically involve the application of an external force to the intact skull without causing skull fracture, hemorrhage, or overt tissue damage acutely. Weight drop (WD) and controlled cortical impact (CCI) are two commonly used models of TBI, which have been adapted to study concussions in rodents (80, 81). In the WD model, a weight is dropped through a tube positioned over the head of an anesthetized animal, to deliver the impact either directly to the head/skull or onto a surface covering the skull (i.e., a metal helmet or gauze) (Table 1). For the modified CCI model, an electronically driven piston impacts the intact skull (Table 2). The head may or may not be stabilized at the time of impact, and models that allow for movement of the head typically produce a diffuse pattern of injury (82). Only models that involve minimally invasive experimental procedures (e.g., no craniotomy) are examined in this review. Experimental concussions vary considerably across several key parameters, such as the weight of the impactor and height from which the weight is dropped for WD models, and the impact velocity, depth, dwell time, and tip diameter for modified CCI models. Regardless of the model employed, the number of impacts, and the time interval between successive impacts in repetitive concussive models are additional determinants of outcome. The high diversity of parameters, as illustrated in Tables 1 and 2, renders direct comparison of findings difficult across different laboratories.

**Table 1:** **Key parameters of weight drop (WD) models of concussive injury**.

Reference	Species and age	Sex	Weight used	Impact location	Skull exposed; head immobile	Drop height	# Impacts	Interval
(83)	Mouse, adult	M	50, 100, or 150 g	Dorsal head	No; no	40 or 60 cm	0, 1, 4	24 h
(84)	Mouse, adult	M	21 g	Dorsal head	No; yes	35 cm	3	24 h
(85)	Rat, adult	M	450 g	Midline	Yes; no	500, 750, or 100 cm	0, 1, 2, 3	1.5, 3, 5, or 10 h
(86)	Mouse, adult	M	95 g + steel cap	Midline	No; no	100 cm	1, 5, 10	24 h
(87)	Mouse, adult	M + F	54 g	Dorsal head	No; no	97 or 107 cm	0, 1, 3, 5, 10	24 h, 1 week, or 1 month
(88)	Mouse, adult	M	95 g	Left parietal	Yes; yes	~10 cm	0, 2	24 h
(89)	Mouse, adult	M	54 g	Dorsal head	No; no	71 cm	0, 1, 5, 7	1 or 2 days; 1, 2, or 4 weeks
(90)	Mouse, adult	M	36.73 g	Left parietal	Yes; N/A	15 cm	0, 1, 2	3 or 20 days
(82)	Mouse, p28	M	95 g + steel cap	Midline	No; no	100 cm	1, 5, 10	24 h
(91)	Mouse, adult	M	54 g	Dorsal head	No; no	71 cm	0, 7	24–48 h
(92)	Mouse, p35–42	M	40 g	Dorsal head	Yes; no	100 cm	1, 4, or 12	1–4 days (1–3/day)

*N/A, not stated in paper; M, male; F, female; p, postnatal day*.

*Gray shading highlights studies which have examined adolescent-aged rodents*.

**Table 2:** **Key parameters of modified controlled cortical impact (CCI) models of concussive injury**.

Reference	Species	Sex	Tip width	Tip material	Impact location	Skull exposed; head immobile	Velocity, depth, dwell time	# Impacts	Interval
(93–95)	Mouse, adult	M	6 mm	Rubber-coated	Left parietal	Yes; yes	4.8–5.6 m/s, 1 mm, n/a	0, 1, 2	24 h
(96)	Mouse, adult	M	9 mm	Silicone	Left parietal	Yes; yes	4.8–5 m/s, 3 mm, n/a	1, 2	3, 5, or 7 days
(97)	Mouse, adult	M	9 mm	Silicone	Left parietal	Yes; yes	4.8–5 m/s, 2 mm, n/a	16	20 min (4 in 1 day), once per week for 4 weeks
(98, 100)	Rat, p35	M	5 mm	N/A	Left parietal	No; no	Head displaced 8 mm at 36 psi	1, 2	1 or 3 days
(101)	Rat, adult	M	N/A	Silicon	N/A	Yes; yes	5 m/s, 3 mm, n/a	1, 2	8 days
(102)	Mouse, adult	M	9 mm	Rubber	Left parietal	Yes; yes	5 m/s, 3.3 mm, 100 ms	1, 2	24 h
(103–105)	Mouse, adult	N/A	9 mm	Rubber	Left parietal	Yes; yes	n/a, 3.3 mm, n/a	0, 2	24 h
(106, 107)	Mouse, adult	M + F	N/A	Metal	Midline	Yes; yes	5 m/s, 1 mm, 200 ms	1, 5	48 h
(108)	Mouse, adult	M	5 mm	Metal	Midline	Yes; no	5 m/s, 1 mm, n/a	1, 4	24 h
(109)	Mouse, adult	M	9 mm	Rubber	Left parietal	No; no	4 m/s, 3 mm, 200 ms	0, 1, 3	24 h
(110)	Mouse, adult	M	6 mm	Rubber	Left parietal	No; no	5 m/s, 1 cm, 100 ms	0, 1, 42	2 h (6 daily hits) for 7 days
(111, 112)	Rat, p35	M	5 mm	N/A	Left parietal	No; no	Head displaced 8 mm at 36 psi	0, 4	1 day

*N/A, not stated in paper; M, male; p, postnatal day*.

*Studies from the same laboratory using similar or identical parameters are grouped together*.

*Gray shading highlights studies which have examined adolescent-aged rodents*.

To date, few laboratories have developed models of concussive-like brain injuries in juvenile animals (98, 113) and of these only one (98) has examined the consequences of repetitive insults. Here, a modified CCI model has been utilized to generate concussions in male rats at postnatal day 35 (p35), estimated to approximate an adolescent-aged animal based upon behavioral and biochemical features at this age (98). In this model, the head is allowed to move freely in the direction of impact, resulting in apnea and delayed righting and toe pinch responses in the absence of a skull fracture. Two or four repeated injuries have also been characterized, with an inter-injury interval of 24 h (98, 100). A second juvenile model, developed by Mychasiuk and colleagues by adapting the Wayne State method (82, 86), has thus far focused on a single concussive-like insult. In this model, p30 rats are positioned onto a piece of aluminum foil – upon impact to the head, the animal undergoes a 180° horizontal rotation to fall freely onto a cushioned surface. These rats also exhibit a delayed righting response immediately after injury (86, 113).

### Behavioral consequences of experimental concussions at adulthood

Studies of the behavioral consequences of concussions in rodents typically focus on cognitive, emotional, and/or sensorimotor functions – processes that may be disrupted or altered following a concussion in humans (15, 16). A commonly used test for cognition in rodents is the Morris water maze (MWM), which specifically evaluates spatial learning and memory [for a detailed description, see Ref. (114)]. In adult animals, these cognitive functions are generally unperturbed by a single concussive injury (93, 101, 113). In contrast, repetitive concussions may produce deficits in spatial learning and memory, which manifest as early as 24 h after injury and may persist for several weeks to months (84, 87, 91, 102, 108). Further, greater cognitive deficits have been reported after a shorter time interval between repetitive concussions (up to 7 days apart) (87, 89, 96). Non-spatial cognitive deficits resulting from concussions have also been demonstrated by the novel object recognition (NOR) test, which evaluates recognition memory (88, 115).

Several studies have also reported concussion-induced balance problems along with numerous other sensorimotor deficits, in experimental models of single and repeated concussions. The accelerating rotarod is the most commonly used tool to evaluate sensorimotor function in rodents (116), and in adult animals, transient motor deficits have been reported within the first week post-injury after repetitive concussive insults, with recovery by several weeks to months later (88, 106, 109). Together, these findings point toward transient motor dysfunction following concussive brain injury, compared to cognitive changes, which may persist for weeks to months after repeated injuries (Table 3). As with cognitive dysfunction, inter-impact interval appears to be a determinant of sensorimotor impairments, whereby shorter intervals lead to greater duration and severity of motor impairments (96).

**Table 3:** **Chronic behavioral deficits detected after repeated concussive injuries in rodents**.

		Time point (months)	# Impacts	Frequency/interval between impacts	Injury model	Test	Reference
Cognitive deficits	Persistence	1	5	1 and 7 days	WD	MWM	(87)
			2	3 days	WD	BM	(90)
			42	2 h (6/day × 7 days)	CCI	MWM	(110)

		1.75	2	1 day	CCI	MWM	(102)

		2	5	1 day	WD	MWM	(89)
			3	1 day	CCI	RAWM	(109)

		3	4	1 day	CCI	APACL	(108)

		6	5	1 and 7 days	WD	MWM	(89)
			42	2 h (6/day × 7 days)	CCI	MWM	(110)
			3	1 day	CCI	FC	(109)

		12	5	1 day	WD	MWM	(89)
			5	1 day	WD	MWM	(87)

	Absence	1	5	30 days	WD	MWM	(87)

		2	2	1 day	CCI	MWM	(93)

		4	2	1 day	CCI	MWM	(94)

		6	16	20 min (4/day, 1 day/week for 4 week)	CCI	MWM	(97)
			5	14 days and 30 days	WD	MWM	(89)
			3	1 day	CCI	YM	(109)

		12	5	7 days	WD	MWM	(87)

Sensorimotor deficits	Persistence	2	2	1 day	CCI	RPT, RR	(93)

	Absence	1	2	1 day	CCI	CN, RPT	(93)
			5	1 day	WD	OF	(86)
			5 or 10	1 day	WD	RR	(82)

		1.5	3	1 day	CCI	SHM, RR	(109)

		4	2	1 day	CCI	CN	(94)

		6	16	20 min (4/day, 1 day/week for 4 week)	CCI	CN	(97)

Depressive-like symptoms, anxiety, and sleepdisorders	Persistence	1	42	2 h (6/day × 7 days)	CCI	EPM, FST, TST, DSI	(110)
			5 or 10	1 day	WD	ST, NSFT	(82)

		6	42	2 h (6/day × 7 days)	CCI	EPM	(110)

	Absence	1.5	3	1 day	CCI	EZM	(109)

*Studies, which conducted behavioral testing at or later than 1 month after CCI or WD injuries, are tabulated, and outcomes categorized as cognitive, sensorimotor/activity, or other (anxiety, depression, and sleep disorders). Together, these studies suggest that cognitive deficits may persist long-term after repeated concussions, compared to sensorimotor dysfunction, which is typically resolved by this time*.

*APACL, active place avoidance and conflict learning; a test for spatial learning, memory, and cognitive flexibility; BM, Barnes maze, a test for spatial learning and memory, and memory retention; CN, composite neuroscore, a test for motor function; DSI, Data Sciences International EEG and EMG recordings; EPM, elevated plus maze, a test for anxiety; EZM, elevated zero maze, a test for anxiety; FC, fear conditioning, a test for contextual associative learning; FST, forced swim test; MWM, Morris water maze, a test for spatial learning, memory, and memory retention; NOR, novel object recognition, a test for recognition memory; NSFT, novelty suppressed feeding test, a test for anxiety; OF, open field, a test for general activity motor function; RAWM, radial arm water maze, a test for spatial learning, memory, and memory retention; RPT, rotating pole test, for motor function and coordination; RR, rotarod, a test for sensorimotor function and learning; SHM, smart home-cage monitoring, which tests for activity and exploratory behavior; ST, splash test, for depressive-like behavior; TST, tail-suspension test, for depressive-like behavior; YM, y-maze, a test for working memory*.

### Behavioral consequences of experimental concussions at adolescence

Cognitive deficits, including impaired recognition memory evident as a decreased preference for a novel object, are visible by 24 h after either a single or two repetitive concussions in male p35 rats (98), but by 3 days, deficits persisted only in those with repeated concussions (100). In contrast, these rats showed normal activity and anxiety levels, performing similarly to sham animals in the open field test at 24 h after surgery (98). However, 10 daily concussions to adolescent (p28) mice impaired balance and coordination on a rotarod at 24 h after injury, which recovered when re-tested 1 month later (82), consistent with evidence that sensorimotor deficits also tend to be transient in nature after repetitive concussions in adults (86).

Given that adolescence is a time of pubertal hormonal changes, which influence brain development, it is possible that concussions may interfere with this maturation process. Indeed, four repetitive concussions (24 h intervals) to p35 male rats results in reduced testosterone production both acutely (24 h) and sub-chronically (2–4 weeks), with recovery to age-appropriate levels by 2–3 months after injury (112). These mice also exhibited a delayed onset of puberty, erectile dysfunction, and abnormalities in reproductive behaviors including social interest and copulatory interactions. The authors link these observations with hypopituitarism, and provide further evidence that repetitive injuries depress growth hormone and insulin-like growth hormone levels at 1–4 weeks after injury, which was associated with reduced weight gain in these animals (111). In light of ongoing controversy on whether or not to test for or treat hormonal deficiencies after TBI in children, these findings suggest that even a transient window of hormonal imbalance due to repetitive concussions at adolescence may result in long-lasting changes to critical adult behaviors.

## Identifying the Unique Pathological “Fingerprints” of Concussive Brain Injuries

We are only just beginning to fully appreciate how concussions may influence the structural and functional integrity of the brain. Here, we briefly review the salient neuropathological features of single and repeated concussive impacts based upon evidence from experimental models in adult and adolescent-aged animals.

### Pathology after experimental concussions at adulthood

In animal models of concussions, acute and chronic pathology is often not readily apparent by traditional methods of detection. A single concussive injury is most often characterized by the lack of skull fracture and absence of acute intra-parenchymal hemorrhage or cerebral contusion (117, 118), and tissue integrity typically appears intact (93, 106–108), even after two repeated concussions (90, 102). Several studies have detected neuronal loss only after repetitive injuries, suggestive of an additive or cumulative effect (93, 108). Evidence of pathology in experimental models is difficult to compare due to differences in injury severity, methodologies and detection methods, and selected time points for analysis. Of note, cognitive deficits are often reported after repetitive concussions to adult rodents in the absence of cell death, suggesting that a lower threshold of injury may be needed to induce functional deficits compared to that required for neuropathological changes (83, 87).

Axonal injury may be detected after even a single concussive insult in the adult rodent brain, often in the absence of overt cell death (106, 108, 118). Widespread silver staining and immuno-reactivity for beta amyloid precursor protein (β-APP), a commonly used indicator of impaired axonal transport, provides evidence of axonal injury as early as 24 h after a single concussion in the adult rodent brain (84, 96, 106, 119), which may persist for up to 2 months (93, 108). Patterns of axonal injury are consistently increased by repetitive brain insults (90, 93, 96, 102, 108). Diffusion tensor imaging in adult mice after repetitive concussions has corroborated these findings of white matter damage (103).

Enhanced glial activation, most commonly detected by immuno-labeling with glial fibrillary acidic protein (GFAP) and ionized calcium binding adaptor molecule-1 (Iba-1), has been associated with regions of axonal injury after even single concussive injury in the adult rodent brain (90, 108, 109, 118). This inflammatory response is exacerbated after repeated insults (90, 102–104, 106, 108), and may persist for several months following the final impact (91, 106). However, as is the case with more severe CNS injury, the precise role of inflammation in neurodegeneration versus repair remains unclear (120). Recent experiments aimed to determine the contribution of CD11b+ microglia to secondary axonal injury processes, by inducing two repetitive injuries (24 h apart) in CD11b-thymidine kinase transgenic mice. Surprisingly, the resulting microglial depletion had little effect on the degree of axonal injury by several complementary markers (105). Thus, the relationship between cerebral inflammation and axonal degeneration after concussions remains unclear.

Several studies have also demonstrated an increase in total or phosphorylated Tau protein after repeated impacts at both sub-acute (30 days) and chronic time points (3–6 months) post-injury (86, 109). However, a considerable number of studies have failed to detect such changes long-term (89, 92, 97, 106), such that the link between repetitive concussions and chronic neuropathology remains inconclusive to date.

### Pathology after experimental concussions at adolescence

Given the unique properties of the brain across development, it is possible that pathogenesis may be influenced by age at the time of concussion. Moreover, repeated concussions across an extended developmental timeline may likewise exhibit distinct pathological changes. However, to date, very limited data are available regarding injuries to the adolescent brain. One study has examined acute pathological markers after two repetitive impacts in the adolescent (p35) rat, reporting no evidence of neuronal degeneration by FluoroJade staining (98). However, an increase in β-APP-labeled axons was evident in the ipsilateral white matter of adolescent rats after repeated (but not single) concussions, alongside an increase in GFAP-positive astrocytes at the gray–white matter junction. Further work is needed to confirm these findings across both an acute and chronic time course, and to determine the threshold (e.g., the number and timing of repetitive concussions) at which neuronal and axonal integrity becomes compromised. Improving our understanding of changes in white matter integrity and connectivity following concussions at adolescence is crucial, particularly in light of recent evidence of white matter abnormalities in high school and college-aged athletes, even in the absence of a clinically diagnosed concussion (23, 121).

### Improving animal models of concussive injuries to the developing brain

While adult rodent models of concussions are at the forefront of investigation, there is opportunity to expand this effort to the adolescent brain. Assessment of age-dependent vulnerability to concussive brain injury remains in its infancy. Functional and pathological outcomes, across both an acute and chronic time course in young animals, may reveal potential vulnerability or resilience during childhood and adolescence. To improve translation, such approaches are predicated upon gaining a better understanding of the respective time courses of rodent versus human brain development and associated behaviors. Current modeling of repetitive concussions to the adolescent brain does not fully encapsulate all populations at risk of experiencing repetitive concussions. For example, in light of epidemiological evidence suggesting poorer outcomes in females after concussions (52, 75) adequate representation of both sexes in animal models is warranted and may in fact identify important sex-specific responses to injury.

Further, there is much to be gained by a critical examination of the current adult models of concussion and their relevance to the concussed human brain. For example, many models involve restriction of head movement via positioning in a fixed stereotaxic frame, contrary to emerging evidence suggesting that head rotation, resulting in acceleration and deceleration forces are necessary components of a concussion (82, 122). Several groups have now modified traditional models to incorporate an element of movement to the head and/or body, or developed new devices to generate clinically relevant brain injuries (123–125). Despite such advances, there remains a lack of consensus regarding the appropriate interval between repetitive injuries to provide the most relevant model of the human condition. Further, it is unclear how loading weights in rodent models compare to injury forces seen in concussed patients, and whether we are approximating an appropriate injury severity in line with what is seen clinically. For example, LOC is a feature of <10% of concussions (4, 17); yet, the surrogate marker in rodents, of prolonged righting time immediately following the impact, remains a salient characteristic of many animal models (80). As findings from experimental studies may inform clinical management decisions, for example, in determining the optimal timing for returning to activity, it is important to consider these limitations and support ongoing efforts to overcome these challenges and improve animal modeling.

## Clinical Management of Concussions – Evidence or Intuition?

While experimental models serve as a foundation for understanding the pathobiology of concussions in children and adults, the questions asked by basic researchers may also be guided by clinical uncertainties regarding optimal medical management. Here, we provide a brief overview of current clinical management, and demonstrate how animal modeling may offer insight into critical questions posed by the medical community.

Clinical management of concussion has predominantly been dictated by expert-derived position statements. These position statements are routinely revisited and released, and are considered a key reference source for medical doctors, athletic trainers, and neuropsychologists, as well as other health-related professionals involved in the treatment of concussed young people (126). Such guidelines have persisted for nearly two decades, and are historically based upon the judgment and clinical intuition of field leaders rather than empirical evidence of physiological data, or an understanding of injury mechanisms (127, 128). Updated concussion guidelines have recently been published by the American Medical Society for Sports Medicine (129), the Concussion in Sports Zurich Consensus Working Group (11), and the American Academy of Neurology (12).

However, there remains a lack of strong empirical evidence to support these guidelines of concussion management, and recommendations continue to be at least partly based upon expert opinion and educated speculation, particularly when considering young athletes. As a consequence, controversy remains regarding what constitutes the “optimal management” of a concussion. The purpose of concussion management is to promote recovery, reduce the risk of recurrent injuries, and minimize the occurrence of long-term symptoms (12). Guidelines emphasize the importance of education for the athlete and their family; support neurocognitive and balance testing using reliable and validated assessments; and advocate for both physical and cognitive rest after concussion. All current guidelines recommend that a concussed athlete be removed from play immediately, and it is generally accepted that an athlete must not return to play (RTP) until concussive symptoms have resolved (11, 130).

### Return to play guidelines

Much of the debate over post-concussive management in both children and adults is focused on RTP. A RTP schedule is typically based upon two yet-to-be-proven concepts: first, that sustaining a single concussion may render the individual more vulnerable to a subsequent injury; and second, that repeated injuries within a short time window may result in cumulative structural and functional consequences. Although not yet definitive, both of these concepts are gaining support from accumulating laboratory, clinical, and epidemiological research.

Return to play decision making by healthcare providers should depend upon close monitoring of symptoms over time, as well as reference to standardized checklists, age-matched normative data, or pre-injury baseline testing. The RTP guidelines presented in the Zurich Concussion in Sports Consensus statement are widely accepted as the current standard of care (11), and consist of a six-step pathway (Table 4). Return to work or study should follow a similar time course (131). It is worth noting, however, that poor neurocognitive performance may persist in concussed college-aged athletes even after concussion-specific symptoms have resolved, cautioning against the exclusive use of symptom resolution for RTP decision making (132).

**Table 4:** **Gradual return to play (RTP) protocol for young athletes**.

	Stage	Functional exercise	Objective
1	No activity	Complete physical and cognitive rest	Recovery
2	Light aerobic exercise	Walking, swimming, or stationary cycling keeping intensity <70% maximum permitted heart rate. No resistance training	Increase heart rate
3	Sport-specific exercise	Skating drills in ice hockey, running drills in soccer. No head impact activities	Add movement
4	Non-contact training drills	Progression to more complex training; e.g., passing drills in football and ice hockey. May start progressive resistance training	Exercise, coordination, and cognitive load
5	Full-contact practice	Following medical clearance participate in normal training activities	Restore confidence and assess functional skills
6	Return to play	Normal game play	Full return to pre-concussion activities

*Adapted from May et al. (133) and Halstead and Walter (39). Each stage requires a minimum of 24 h duration, and athletes are recommended to remain asymptomatic before progression to the next step (11, 79). Once all steps have been completed without symptom exacerbation, the individual may be cleared for full return to athletics participation*.

Athletes at highest risk for sustaining a concussion are those with a history of concussive injuries, and this risk is greatest during the first 10 days post-injury (12, 47). Therefore, RTP decisions initially after injury may impact an athletes’ likelihood of sustaining a repeated head insult. RTP protocols are further complicated for patients who experience repetitive concussive injuries; in these cases, the RTP time course is typically extended to err on the side of caution. RTP protocols have been widely adopted by individual sporting organizations such as the National Football League, as well as state legislation. However, whether implementation of RTP guidelines improves symptom resolution or prevents subsequent concussions has not yet been established. Well-designed studies are required to determine whether current RTP recommendations are the most appropriate (134, 135). Further, the precise definition of being “asymptomatic” for RTP has been questioned, particularly when dependent upon self-reporting of symptoms by young athletes, and in light of reports of post-concussive symptoms in non-concussed populations (136, 137).

### RTP in adolescent athletes

Due to both the paucity of data specifically regarding injury at adolescence and the high degree of diversity between different experimental models, we do not yet have a good understanding of how repeated brain insults may affect the developing brain (47). As such, there are no universally accepted, evidence-based guidelines that have been validated for safe RTP following sport-related concussion in children or adolescents (134, 138, 139). It is generally recommended that child and adolescent athletes never return to physical activity on the same day of injury, and only return when clinically symptom-free or cleared to RTP by a licensed health care professional trained in the evaluation and management of concussions (11, 39, 133).

Typically, a more conservative approach is recommended in children and adolescents compared to adults (130, 140). Of note, a recent study suggested that a minimum of 14 days is required following symptom resolution and normalization of neuropsychological tests before RTP in young concussed athletes, compared to the 7-day protocol typically used for adults (141), based on reports of prolonged neuropsychological impairments in children (6, 7, 79). As with adults, some have advocated for individualized RTP protocols for child athletes, which take into account their unique, sports-specific injury profile, and developmental stage (39, 133).

A unique, under-studied consideration of concussions during childhood and adolescence is the timing of returning to school work or “return-to-learn” (140). As with RTP, a step-wise procedure has been recommended for concussed adolescents returning to school, involving sequential stages of cognitive challenges interspersed with rest periods, which are closely monitored for symptom exacerbation (142, 143) (Table 5). Additionally, the optimal timing for resumption of learning to drive, a newly acquired, cognitively demanding task for older adolescents, should also be carefully evaluated.

**Table 5:** **Gradual return-to-learn (RTL) protocol for young athletes**.

	Stage	Functional exercise	Objective
1	No activity	Complete cognitive rest	Recovery
2	Minor cognitive activity at home	Short periods (5–15 min) of cognitive activity (homework)	Gradual, closely monitored increase in sub-symptom threshold activities
3	Moderate cognitive activity at home	Longer periods (20–30 min) of cognitive activity (homework)	Increase cognitive stamina; self-paced activity
4	Partial school re-entry	Part day of school attendance, plus 1–2 cumulative hours of homework	Re-entry into school with accommodations to maintain cognitive load below symptom threshold
5	Gradual reintegration to school	Gradual increase to full day of school attendance	Increase cognitive stamina; gradual decrease of accommodations
6	Full cognitive workload resumed	Catch up on essential missed work; re-introduce testing and assessments	Full return to school; recommended to commence RTP protocol

*Adapted from Master et al. (131, 142)*.

### Justification for rest: Metabolic and neurochemical dysfunction after concussion

One of the key pathophysiological phenomena characterizing TBI in rodent models is the adverse “neurometabolic cascade,” indicating alterations in cerebral metabolism and blood flow, which may result in an energy crisis (99, 120, 144, 145). Although more commonly associated with moderate-to-severe TBI, changes in cerebral metabolism have also been implicated in the pathophysiology of concussions in both animal models and humans. Such findings are important for identifying a window of vulnerability to repetitive insults, and may prove critical to the establishment of evidence-based guidelines for optimal concussion management.

Evidence from adult animals suggests that the interval between repetitive concussions may determine the extent of metabolic depression – for example, concussions delivered 3 days apart produced a prolonged reduction in key energy metabolites compared to a single insult, or two injuries delivered 5 days apart (146, 147). These data indicate a temporal window of impaired cellular metabolism including mitochondrial dysfunction and oxidative stress. It has been postulated that transient disruptions of cerebral metabolism by concussions may have greater ramifications in the context of the developing brain, where neurochemical alterations may result in disrupted brain plasticity (144, 145). There are known differences in baseline cerebral blood flow and glucose uptake between pediatric and adult populations (10, 148–150), and rodent studies have indicated that even a mild injury can generate hypotension in young rats (144). This is exemplified in studies by Prins and colleagues, who reported a 20% reduction in glucose metabolic rate by 24 h after a single concussion to p35 rats, which was restored to normal levels by 3 days. When a second injury was introduced during this period of metabolic depression, glucose metabolism was reduced even further (~35%) and remained depressed for up to 5 days after the final injury (100). These experimental data demonstrate a critical window of vulnerability to glucose hypo-metabolism after concussive injury, when a second concussion may exacerbate the first. This evidence, together with clinical findings, also suggest that changes in glucose metabolism may resolve more rapidly in younger animals, indicating an age-dependent difference in the duration of metabolic depression post-injury, and emphasizing the need for further age-specific studies (145).

A series of clinical studies have detected similar neurometabolic dysfunction after concussions in adult humans, which are typically longer lasting compared to rodents (144). Using proton magnetic resonance spectroscopy, repeated concussions were associated with mitochondrial malfunction and a prolonged time to normalization of energy metabolites to 15 days (151). Interestingly, the time course of metabolic recovery after even a single concussion extended past the resolution of post-concussive symptoms, prompting concern that being “symptom-free” may not necessarily imply a return to normal brain homeostasis (34, 36). In children and adolescents, a significant depression of cerebral blood flow was identified in concussed individuals aged 11–15 years, which persisted beyond 30 days after injury in the absence of neuronal or axonal disruption by MRI (35). Corroborating this finding, reduced cerebral blood flow was also demonstrated in a small cohort of concussed patients aged 5–17 years, as well as regional changes in neuronal metabolites including *N*-acetyl aspartate (152).

### Is Rest the Best Medicine? The Clinical Evidence

Current guidelines for concussion management in sports advocate for physical and cognitive rest after injury (72, 129), which is argued to be the best immediate treatment for concussion (153). Physical rest refers to the absence of athletic activities, as well as a reduction in daily life activities that require exertion, such as trips and social visits in or out of the home (12, 39, 154). Cognitive rest involves minimizing activities that require high levels of concentration and attention, including reading, board games, television, text messaging, computer, and video games (153, 154). For younger athletes, this definition should extend to include homework and academic workloads (131). The assumption is that symptom exacerbation due to physical or cognitive activity is a sign that brain activity is triggering neurometabolic dysfunction (131). However, empirical evidence attributing efficacy to post-injury rest is limited, and high-quality studies are needed to evaluate this commonplace intervention. Second, the definition of what exactly constitutes “rest” varies considerably between studies, and both physical and cognitive rest are often evaluated together. Further, the optimal timing of reintegration into activity following a period of rest has not been established. Of those studies that have aimed to test the efficacy of rest after concussion, it is commonly difficult to distinguish between a change in status attributed to rest compared to the normal trajectory of recovery over time. Key studies examining the potential effects of rest after concussion are summarized in Table 6. Most recently, a cohort of concussed adolescents were randomized to either 5 days of strict rest or standard care (1–2 days of rest followed by graded RTP) (20). The groups did not differ in terms of neurocognitive or balance outcomes; however, the “strict rest” group reported more symptoms and slower symptom resolution across a 10 days period.

**Table 6:** **Clinical evidence regarding rest after concussions**.

Reference	Cohort	Intervention	Key findings	Caveats
(155)	54 Patients with “mild TBI” in emergency room	±complete bed rest for 6 days	• Both groups showed improvement over time	• Cohort consists of more severe injuries than sports-related concussions
			• Complete rest did not alter the recovery rate	
(156)	95 High-school athletes	Self-reported “activity intensity scale”	• Engagement in highest intensity of activities exhibited greatest impairments in visual memory and reaction speeds	• Potential for recall bias when self-reporting activity levels
(157)	635 High school and college-aged athletes	Prospective; “symptom- free waiting period” or not prior to RTP	• No effect of post-injury rest on symptom recovery, neuropsychological, or balance testing, or risk of a repeat injury	• Lack of randomization
(158)	184 Athletes (8–26 years old)	Cohort study; ±recommended cognitive rest	• Longer duration of symptoms in individuals who were recommended rest	• Individuals prescribed rest may have had a more severe injury
(159)	49 High school and college-aged athletes	≥1 week physical and cognitive rest, followed by graded reintegration of activity	• All demonstrated involved neurocognitive performance and reduced symptoms	• Lack of blinding, randomization or a control group
			• Longer period of rest was associated with more improved cognitive processing speeds	
(33)	335, 8–23 years olds reporting to a concussion clinic	Prospective; self-reported cognitive intensity survey	• Highest cognitive activity post-injury associated with a longer duration of symptoms	• Potential for recall bias when self-reporting activity levels
(160)	13 Adolescents (subset of 2012 study with persistent symptoms)	Prescribed 1 week of “comprehensive rest”	• Improved symptoms in 62% of athletes	• Lack of randomization or a control group
				• Potential contribution of spontaneous recovery and education
(20)	99, 11–22 years olds presenting to a pediatric ED	Prospective; randomized to strict rest for 5 days or usual care (1–2 days rest then step-wise RTP)	• No difference in neurocognitive or balance outcomes at 3 or 10 days	• Lack of blinding
			• Group assigned strict rest reported more symptoms and slower resolution at 10 days	• Potential for recall bias when reporting symptoms

Given the limited studies in this arena, and their inconclusive nature, the IOM’s recent report titled “Sports-Related Concussions in Youth” stated that only weak evidence exists in support of physical and cognitive rest for adolescents following concussion (10). Similarly, the 2013 AAN guidelines, which specifically focused on formulating evidence-based recommendations, noted that “no conclusions can be drawn regarding the effect of post-concussive activity level on the recovery from sports-related concussion or the likelihood of developing chronic post-concussion complications”(12).

## Activity-Based Rehabilitation – Incorporating Exercise in Concussion Management?

With guidelines for RTP after mild TBI now legislated in state law across the US, it is imperative that effective, evidence-based treatment strategies are established. While management guidelines typically call for physical and cognitive rest to avoid aggravation of symptoms, a paucity of evidence exists to support the presumed benefits of rest. On the contrary, physical and cognitive training are the foundation of rehabilitation for more severe TBI (161), raising the question of whether mild activity might not only be tolerated after concussion but may also promote better recovery for patients with persistent symptoms (162–164).

### Delayed onset exercise promotes recovery after CNS injury in experimental models

The benefits of physical exercise have been studied in injured or diseased CNS. For example, exercise training in rodents after CNS injury promotes neural plasticity by increasing neurotransmitters, neurotrophic factors, and neurogenesis (165–169). In both animal models and patients with neurodegenerative conditions such as multiple sclerosis and Huntington’s disease, cognitive stimulation also induces trophic factors and may provide neuroprotection, benefiting long-term cognition and quality of life (170–172). In particular, brain derived neurotrophic factor (BDNF) is highly responsive to activity, increasing in response to wheel running, environmental enrichment, and task-specific practice following brain injury (166, 173). BDNF appears to be important for synaptic plasticity, and likely plays a role in improved motor and cognitive function by re-establishing dense synaptic networks after various CNS injuries (165, 169).

As demonstrated in animal models of adult TBI, and reviewed elsewhere (174), the effects of exercise after moderate-to-severe injuries vary depending upon the timing, intensity, and voluntary nature of the activity. In general, sub-acute or chronic exercise yields neuroprotection (166, 168, 175, 176), whereas implementation of exercise during the early post-injury period is largely detrimental to cognitive performance (166, 177, 178). Together, these findings are in accordance with the hypothesis that early onset exercise introduces an increase in metabolic demand at a time when the brain is energetically compromised, with detrimental consequences on functional recovery (178). However, the onset of carefully monitored exercise interventions during the sub-acute or chronic phases of CNS injury may promote mechanisms of recovery. Of note, studies to date have focused exclusively on moderate-to-severe TBI in adult animals – thus, the effect of exercise after single or repetitive concussive brain injuries has yet to be studied in the adult or the adolescent brain.

### Are there risks associated with “rest” after concussion?

The risk of causing damage with early exercise following trauma is certainly valid, based upon evidence from experimental models of CNS injury that exercise initiated early or during periods of high inflammation results in detrimental cellular and behavioral consequences (166, 168, 179–181). Using rest to promote recovery from CNS injury would thus seem to be a conservative and safe approach; however, imposing rest after concussion in a previously active athlete may result in the withdrawal of growth and trophic factors at the site of injury. When rats that were habitual runners are deprived of exercise, brain expression of BNDF drops well below levels in normally sedentary rats (182). Translating this finding to brain-injured humans, the imposition of rest may create a situation where BDNF expression is critically low after injury, resulting in an environment conducive to greater neural damage and impaired neuroplasticity. Moreover, regular exercise in non-injured populations has been shown to minimize depression, which can be a serious consequence of even mild TBI (183, 184). Thus, it is possible that the withdrawal of physical activity post-injury may compound or contribute to the manifestation of injury-induced depression (163). Further research is needed to evaluate the effects of activity-based rehabilitation on psychiatric outcomes after concussive brain injuries.

### Activity-based rehabilitation after repetitive concussions

Both physical and cognitive rehabilitation are considered integral to promoting recovery after moderate-to-severe TBI (163, 185). Vestibular rehabilitation has proven beneficial for patients presenting with persistent dizziness and balance deficits, by focusing on restoring the vestibular system’s ability to adequately detect motion of the head, and in turn maintain postural control and stability in patients (186, 187). Some evidence also supports the utility of vestibular rehabilitation in patients younger than 18 years (188, 189), particularly for persistent symptoms. In concussion, preliminary studies have suggested that physical exercise is both safe and has positive effects on brain recovery during the sub-acute post-injury phase in children and adolescents (190, 191). In both of these studies, exercise training was carefully monitored to ensure it remained submaximal (below the threshold for symptom exacerbation), avoiding the postulated negative outcomes associated with excessive exertion.

In line with this concept, evidence has emerged suggesting that low-to-moderate activity may provide benefit in adolescent patients with symptoms after concussion. Majerske and colleagues found that, although high levels of physical activity were associated with poor outcomes, moderate levels of exertion (defined as “*light activity at home*” and “*school activity*”) were in fact associated with better outcomes compared to those who rested completely or participated in only cognitive activity (156). In a separate study, although the highest level of cognitive activity was associated with a longer duration of symptoms after concussion, patients who engaged in all other levels of cognitive activity (from “*complete cognitive rest*” up to “*significant cognitive activity, but reduced from that you would usually do*”) had a similar trajectory of symptom duration (33). Together, these studies suggest that the complete abstinence of physical and cognitive activity may be unnecessary and in fact sub-optimal for recovery after concussions. Further, moderate levels of activity may in fact promote recovery of persistent symptoms.

Although there are no randomized controlled trials to guide the resumption of activity after concussions, the graded incorporation of exercise and cognitive activities may be of use in improving outcomes after concussions, and future studies to investigate the timing and efficacy of such interventions are warranted (163). One possible approach would be to combine light physical and cognitive activity during the sub-acute to chronic time period with the objectives of expediting recovery, addressing cognitive impairments, and attenuating long-term sequelae after TBI. Such an approach would warrant caution, as exercise in the setting of uncontrolled or worsening symptoms may contribute to a neurotoxic environment that interferes with recovery and/or impedes brain development. Recent clinical evidence suggests that performing dual-tasks, typically motor and cognitive activities simultaneously, is well-tolerated by patients (192) and improves both functional and cognitive performance after both mild and severe TBI (161, 193). When treating patients with concussive symptoms, physical therapists may consider introducing activities that have reported good tolerance after severe TBI, such as combining light physical tasks (e.g., walking) with cognitive tasks (e.g., memory tasks).

## Conclusion

The past decade has seen a flourishing increase in attention to the consequences of sports-related concussions, and substantial international efforts to develop guidelines for concussion management. However, evidence supporting such guidelines remains scarce, and further research in both the clinical and experimental arenas is needed to ensure that recommendations align with the best possible outcomes. Importantly, it is becoming evident that concussions in young athletes may differ from those in adult populations in terms of incidence, symptom duration, and risk of recurrent injury, implicating developmentally related susceptibility to mild brain injuries during this crucial stage of ongoing brain maturation. Thus, guidelines developed for adult athletes may not necessarily translate to best clinical practice in children and adolescence. Much work remains to better understand the impact of repetitive concussions in youth, and animal models can provide the opportunity to delineate both the biomechanical and pathophysiological mechanisms, which underlie the manifestation of post-concussive symptoms. However, there remains a concerning disparity between experimental models and the clinical scenario, which may be addressed by improving the alignment between experimental and clinical studies in this field in terms of subject choice, injury mechanisms, and clinically relevant outcome measures. Lastly, the paucity of empirical evidence supporting physical and cognitive rest after concussive brain injuries has been identified, particularly in the adolescent. While caution is warranted to ensure that activity is delivered within symptom tolerance, carefully timed and monitored mild-to-moderate exercise may offer an untapped potential to enhance recovery after repetitive concussions in adolescent athletes.

## Author Contributions

BS, LN, JB, and DF for design and synthesis; SL for pathology; BS, RS, JC, and MP for experimental models and behavior; BS, NF, CG, VH, DB, and LN for rehabilitation; BS, VH, PJ, AL, BP, SS, and MP epidemiology, etiology, and current guidelines.

## Conflict of Interest Statement

The Review Editor Vassilis E. Koliatsos declares that, despite being affiliated to the same institution as author Nora Fritz, the review process was handled objectively and no conflict of interest exists. The authors declare that the research was conducted in the absence of any commercial or financial relationships that could be construed as a potential conflict of interest.
